# Urban Care for Unpaid Caregivers: Community Voices in the Care Block Program, in Bogotá, Colombia

**DOI:** 10.1007/s11524-024-00899-z

**Published:** 2024-09-24

**Authors:** Paula Guevara-Aladino, Olga L. Sarmiento, María Alejandra Rubio, Lina María Gómez-García, Zakaria Nadeem Doueiri, Diego Martínez, Abby C. King, Adriana Hurtado-Tarazona, Ann Banchoff, Luis A. Guzman, María José Álvarez-Rivadulla, Leonardo Palencia

**Affiliations:** 1 School of Medicine, Universidad de los Andes, Bogotá, Colombia; 2 Department of Epidemiology & Population Health, Stanford University School of Medicine, Stanford, CA USA; 3 Grupo de Sostenibilidad Urbana y Regional SUR, Department of Civil and Environmental Engineering, Universidad de los Andes, Bogotá, Colombia; 4 Interdisciplinary Center for Development Studies, Universidad de los Andes, Bogotá, Colombia; 5 School of Social Science, Universidad de los Andes, Bogotá, Colombia; 6 School of Engineering, Universidad de los Andes, Bogota, Colombia; 7 Stanford University School of Medicine (Stanford Prevention Research Center), Stanford, CA USA; 8 Our Voice Global Citizen Science Research Initiative, Stanford, CA USA

**Keywords:** Care, Unpaid caregivers, Urban planning, Care policy, Infrastructures of care, Citizen science, Community-based participatory research, Our Voice, Latin America

## Abstract

The Care Block of Bogotá, Colombia, is an urban program that offers services for low-income unpaid caregivers. This study aimed to (i) characterize unpaid caregivers’ subjective well-being, mental health symptoms, physical activity levels, and use of public spaces linked to the Care Block; (ii) identify caregivers’ perceived built and social environment facilitators and barriers to accessing the Care Block facility; and (iii) document the community-led advocacy process to improve the Care Block program. The quantitative component included a subjective well-being and mental health symptoms survey, and the System for Observing Play and Recreation in Communities (SOPARC) instrument. The qualitative component included the *Our Voice* citizen science method augmented with portable virtual reality equipment to engage participants in advocacy for changes. Participants (median age of 53 years) dedicated a median of 13.8 h a day to unpaid caregiving, had an average subjective well-being score of 7.0, and 19.1% and 23.8% reported having depression and generalized anxiety symptoms respectively. Caregivers reported that the program fosters their perception of purpose, enjoyment, resilience, and cognitive and emotional awareness. SOPARC evaluation showed that most women engaged in moderate to vigorous physical activity. The caregivers highlighted education, physical activity services, and integration of facilities as facilitators to accessing the Care Block program. Poor quality and lack of sidewalks and roads, limited personal safety, and the risk of pedestrian-vehicle collisions were identified as barriers. Virtual Reality sparked compelling dialogue between participants and stakeholders, allowing stakeholders to reflect on an urban program facilitating unpaid care work.

**Supplementary Information:**

The online version contains supplementary material available at 10.1007/s11524-024-00899-z.

## Introduction

Unpaid care work, which refers to personal care, housework, and the protection of family members, is essential for society’s well-being [1]. However, evidence from high-income countries links the type and time spent on caregiving with negative effects on health and well-being [2]. Unequal gender patterns devoted to unpaid care work cut across geographic regions [3]. Women, compared to men, on average perform more than three-quarters of the total time spent on unpaid care work [3]. On average, women dedicate 4 h and 37 min per day to unpaid care, in comparison to men’s average of 1 h and 51 min per day [3]. In Latin America, the care-related gender discrepancies are even larger [3].

The United Nations’ 5th Sustainable Development Goal on gender equity has called attention to reducing unpaid care work inequalities through high-level interventions that enhance care need co-responsibility between communities and local governments [4]. In Latin America, several countries have implemented relevant care work policies, including the National Integrated Care System in Uruguay, the National Care Policy 2021–2031 in Costa Rica, a Social Registry for caregivers in Chile, and the District Care System in Bogotá, Colombia [5].

The Care System introduced by the District Secretariat for Women in Bogotá, which is now linked within the city’s Master Plan, builds on the premise that transforming cities may contribute to the recognition, reduction, and redistribution of care work [6, 7]. Part of the Care System is the “Care Blocks” program (in Spanish, “Manzanas del Cuidado”), consisting of hubs where unpaid caregivers and their dependents (children, elderly, people with disabilities) can access services (e.g., day-care centers, community centers, supermarkets, schools, laundries, recreational venues, and cultural facilities) within a 20-min walk [6].

The Care Blocks are strategically placed in new and existing infrastructure and intersectoral public services [6]. The model is designed to support caregivers in their personal development via education programs, psychosocial support, physical activity classes, and wellness courses [6]. The program also implements workshops focused on fostering cultural change to reduce care-related inequities [8]. Additionally, care recipients can access care services and recreational activities [7]. In 2023, there were 20 Care Block facilities and by the year 2035, this number is projected to rise to 45 [7]. Currently, around 233 thousand women have received support from the Care Block program.

This current mixed methods study aims to (i) characterize unpaid caregivers’ subjective well-being, mental health symptoms, physical activity levels, and the use of public spaces linked to the Care Block; (ii) identify caregivers perceived built and social environment facilitators and barriers of access and use of the Care Block facility; and (iii) document a community-led advocacy process to further advance the potential benefits of the Care Block program. This study is part of the TrUST study (Urban Transformations and Health: The Case of TransMiCable) from The SALURBAL project (Urban Health in Latin America) [9]. Documenting the multi-level experiences of unpaid caregivers and gathering stakeholders’ perceptions can be key to understanding the most effective ways to address community inequities [10–12].

## Materials and Methods

### Study Setting

**Fig. 1:**
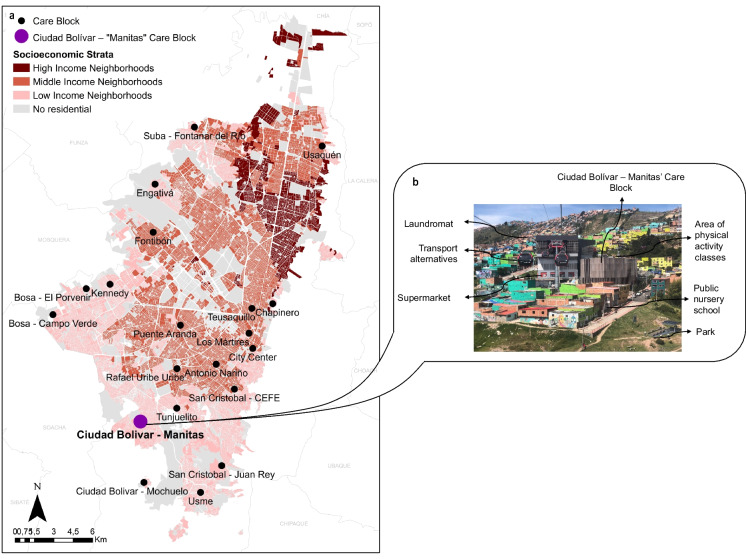
Map of Care Blocks in Bogotá, Colombia, and offered infrastructure in Ciudad Bolivar – Manitas’ Care Block. **a** depicts the geographic distribution of Care Blocks in Bogotá, Colombia, based on neighborhood income. Note that all Care Blocks are located in low-income neighborhoods. **b** depicts a picture of Ciudad Bolivar – Manitas Care Block with the offered services or infrastructure

This study was conducted in Bogotá, Colombia’s capital, which has 7.9 million inhabitants, 52.1% are women [13]. In 2021, 35.4% of women in Bogotá spent over 5 h a day on unpaid care work compared to 8.6% of men [14]. The Care Blocks are located in neighborhoods with the highest concentration of unpaid caregivers, care recipients, and poverty rates (Fig. 1) [7]. In October 2020, the first Care Block was opened in Ciudad Bolívar, an administrative area with 656,015 inhabitants, of whom 10.9% live in multidimensional poverty, and 8.03% are victims of the Colombian armed conflict [15]. Women living in this area face the feminization of poverty and high rates of gender-based violence [16].

Ciudad Bolivar’s Care Block provides the following services: (i) high school curricula, tertiary education, entrepreneurship classes, and career counseling; (ii) psychosocial and self-care programs; (iii) health promotion services including therapy, family medicine appointments, and physical activity classes; (iv) a laundromat; and (v) care training for the broader community (Fig. 1) [6].

### Study Design

**Fig. 2:**
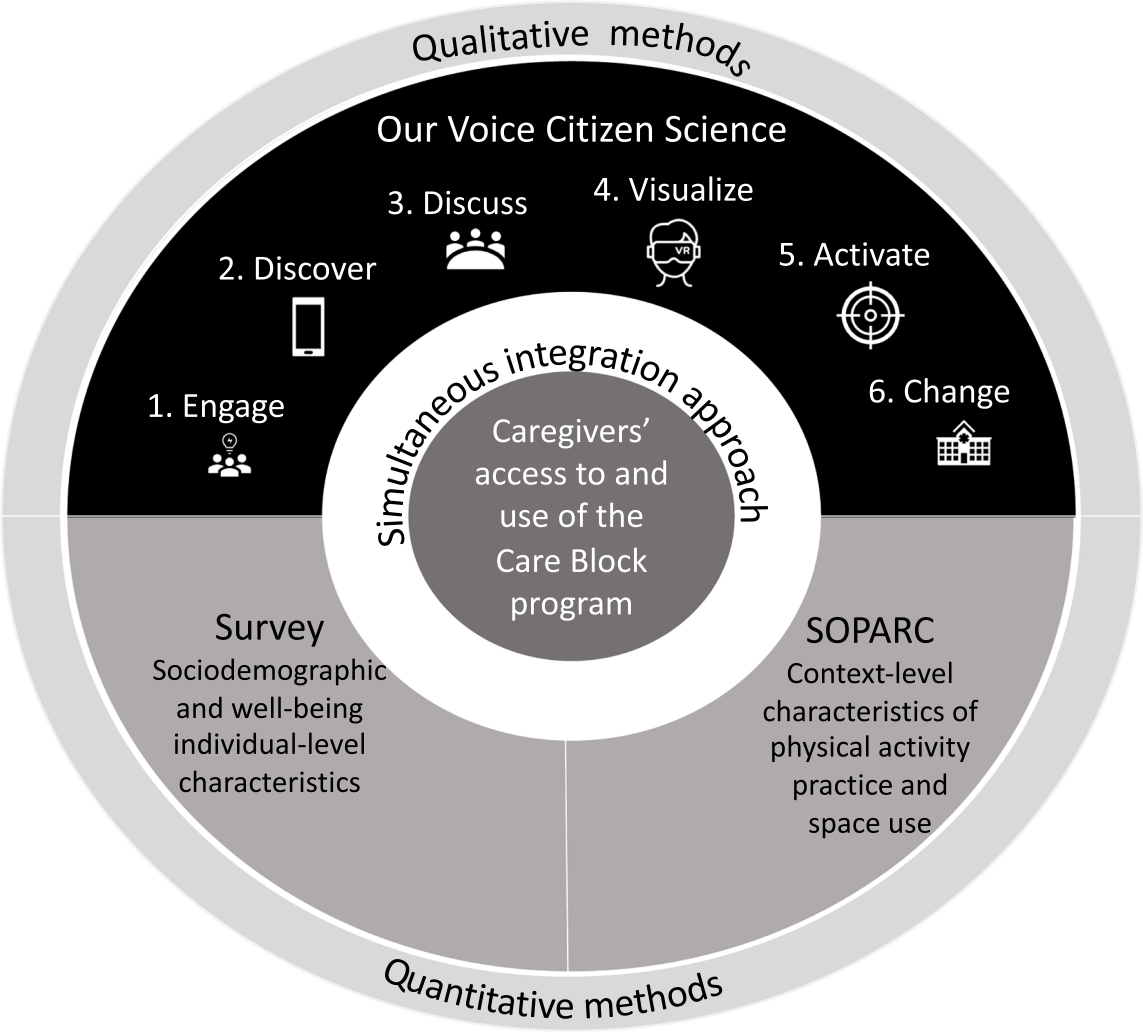
Methodological approach to characterize caregivers’ access to and use of the Care Block program in Bogota, Colombia. This figure depicts the relationship between the multiple methods, altogether aimed at facilitating the understanding of caregivers’ access and use to the Care Block program

This mixed methods study used a simultaneous integration approach [17], within a socio-ecological framework [18], to document the multi-level perceptions of caregivers and stakeholders (e.g., policymakers, implementers, and researchers). The quantitative component included a survey and contextually based systematic observational measurements in the Care Block facility terrace. The qualitative component included the *Our Voice* community-based citizen science method [9], in which we incorporated a virtual reality visualization tool to facilitate discussions regarding recommendations for change. The methods and data harmonization are illustrated in Fig. 2.

### Survey

A convenience sample of participants completed two surveys; the first, implemented between June 24 and July 11, 2022, included sociodemographic characteristics (participants’ age, sex, education, and household socioeconomic status) and care-related information (caregiving time, demographics of dependents, and Care Block use). The second survey was conducted from November 18th to November 22nd, 2022. In this survey, we measured participants’ subjective well-being and mental health symptoms.

Subjective well-being was evaluated using a core question on life satisfaction from the Organization for Economic Co-operation and Development’s (OECD) subjective well-being questionnaire [19]. OECD defines subjective well-being as a personal perception that involves a reflective evaluation of a person’s life or specific circumstances, pleasant or unpleasant emotional experiences, and personal development [19]. Responses to this question are rated on a scale of 1–10 where higher scores indicate higher life satisfaction.

Mental health symptoms (depression and generalized anxiety) were measured using the PHQ-4 questionnaire [20]. Depression symptoms included anhedonia and hopelessness [20]. Generalized anxiety symptoms included emotional and cognitive experiences of generalized anxiety in the last 2 weeks [20]. The questionnaire generates two domain scores (depression [PHQ-2] and generalized anxiety [GAD-2]), each rated on a scale of 0–6 where scores equal to or higher than 3 indicate symptoms of depression or generalized anxiety. For the statistical analysis, we included descriptive statistics (mean, standard deviation, and percentages).

### System for Observing Play and Recreation in Communities (SOPARC)

From December 13 to December 18, 2021, we used SOPARC [21] to assess physical activity levels on a terrace of the Care Block facility where physical activity classes were offered. The terrace was divided into 8 target areas. Two 20-min observations were made on a non-PA class day, and during physical activity classes, four 15-min observation periods were conducted. These observation periods took place 10-min before the class, 10-min and 40-min following the beginning of the class, and 10-min after the class finished. The data collected included the number of users according to sex (man, woman), age group (children, adolescents, adults, or older adults), physical activity levels (sedentary, moderate, or vigorous), and area conditions (accessible, usable, equipped, supervised, organized, or empty). The quality of the Care Block facility was assessed with the Physical Activity Resource Assessment [22]. Comparisons between categorical variables in the user characteristics were tested with a chi-squared test.

### Our Voice Citizen Science Method

*Our Voice* engages community residents as citizen scientists in identifying and changing features of their local environments [10]. From September 2021 to May 2023, we expanded on the standard 4-step *Our Voice* method (discover, discuss, activate, and change) by adding two steps: “engage” and “visualize.” Using the six-step approach, caregivers identified the built and social environmental facilitators and barriers to accessing the Care Block facility, along with activating feasible steps to foster positive change.

### Engage

On September 18, 2021, we conducted a focus group with caregivers to discuss their perceptions and experiences regarding the Care Block program. The focus group was co-created with Bogotá’s District Secretariat for Women. The session consisted of three steps: (i) the attendees rotated through 5 thematic tables facilitated by project members to identify their perceptions and experiences about Ciudad Bolívar’s Care Block; (ii) the group reconvened, and each project member reported the insights discussed at their table while the others simultaneously mapped the insights using the XMind program [23]; and (iii) the group discussed and adjusted the diagrams.

### Discover

In June 2022, caregivers performed community walks to identify local facilitators and barriers to access the Care Block. Community walks were implemented using the Discovery Tool mobile application, which allows participants to capture geocoded photographs and audio/text narratives of features during their journey from their homes to the Care Block facility and vice-versa.

### Discuss

We facilitated two community meetings. In the first one, conducted on July 16th, 2022, citizen scientists reviewed and organized their Discovery Tool data on a whiteboard, identified common themes, collectively prioritized issues, and suggested potential solutions. In the second one, conducted on October 3th, 2022, participants engaged in role-play activities to simulate an experience with stakeholders and then chose five representatives to participate in the subsequent *Visualize* step.

### Visualize

We developed a Virtual Reality Experience (VRE) to share the results and leverage community advocacy with stakeholders by immersing them in a simulation of visual and auditory caregiver walking experiences. The VRE captured five travel experiences to the Care Block that reflected the most mentioned themes. Research staff then returned to these hotspot areas to gather GoPro Max™ 360-degree footage and ambient sound recordings. The *Our Voice* audio recordings were then overlaid onto the GoPro footage with an accompanying voice-over that introduced each participant. The experience was designed using the Unity™ game engine [24]. On February 9, 2023, five citizen scientists tested the VRE using the Oculus Quest 2™ virtual reality headset and were trained to guide the stakeholders on using the immersive headset.

### Activate

On March 9, 2023, citizen scientists and stakeholders participated in a 2.5-h community meeting designed to identify feasible actions to improve access to Ciudad Bolivar’s Care Block. During the session, participants guided stakeholders through the VRE. Simultaneously, all meeting attendees viewed the projections of the immersive headset on a large screen. Then, the group shared their most relevant concerns with the policymakers and proposed solutions to those issues. At the end of the meeting, attendees responded to a virtual reality perception survey.

### Change

Between May 16 and June 5, 2023, we handed citizen scientists an informational guide detailing web page services and trained one caregiver to navigate the informational resource, enabling her to explain it to the rest of the group. Moreover, we held in-person and virtual meetings to disseminate our results with different stakeholders: on May 4, 2023, independently with policymakers from the District Secretariat of Planning, Women, and Habitat; on May 16, 2023, with one citizen scientist; and on May 30, 2023, with a policymaker from Bogota’s Ciudad Bolivar Local Mayors’ Office. Additionally, between May 31 and June 2, 2023, we attended the Smart Cities Expo Bogotá 2023 to disseminate results.

### Analysis

Applying the multi-level socio-ecological framework [18], the Discovery Tool data were analyzed using a content analysis approach and intercoder reliability to characterize themes at the individual, interpersonal, community, policy, and environment levels.

## Results

### Sociodemographic Characteristics

The citizen scientists comprised of 20 women and 1 man, with a median age of 53 (IQR = 40–63). Among them, 42.9% had finished high school and 19.0% had professional, technical, or technological training. Moreover, 66.7% were the heads of their households. They reported dedicating a median of 13.8 h a day to unpaid caregiving (IQR = 9.0–15.8) and had a median of 2 dependents (IQR = 1–3); 52.4% were children, 38.1% elderly, and 33.3% people with disabilities (Table 1).

**Table 1:** Caregiver citizen scientist sociodemographics and subjective well-being, health-related quality of life and mental health symptoms

Variable	*N*	% (if applicable)
*Sociodemographics*		
Number of participants	21	
Age (median [IQR])	53.0 [40.0–63.0]	
Last completed level of education (% out of 21 participants)		
None	2	9.5%
Primary school	6	28.6%
Secondary school	9	42.9%
Technical – college degree	4	19.0%
Relationship to the head of the household (% out of 21 participants)		
Head of the household	14	66.7%
Wife/partner	5	23.8%
Daughter/stepdaughter	1	4.7%
Mother/mother-in-law	1	4.7%
*Care related*		
Daily hours of unpaid caregiving (median [IQR])	13.8 [9.0–15.8]	
Number of care dependents (median [IQR])	2.0 [1.0–3.0]	
Care-receiver group (% out of 21 participants)^1^		
Children	11	52.4%
Elderly	8	38.1%
People with disabilities	7	33.3%
*Care Block services and attendance*		
Services they attend to in the Care Block^1^ (% out of 21 participants)		
Education	18	85.7%
Physical activity and psychosocial orientation	7	33.3%
Spaces for socializing with fellow caregivers	3	14.3%
Care recipient care services	2	9.5%
Transport mode to the Care Block (% out of 21 participants)		
Walking	9	42.9%
Public transport	6	28.6%
Informal transport	2	9.5%
Non-reported	3	14.3%
*Subjective well-being, Mental health symptoms*		
Life satisfaction (average $\pm$ SD)^2^	7.0 $\pm$ 2.2	
Symptoms of depression (% out of 21 participants)^3^	4	19.0%
Symptoms of anxiety (% out of 21 participants)^3^	5	23.8%

*IQR*, interquartile range; *SD*, standard deviation

^1^Percentages do not sum up to 100% because categories are not mutually exclusive (e.g., one caregiver can take care of children and people with disabilities at the same time)

^2^1–10 satisfaction scales (1 = extremely unsatisfied, 10 = extremely satisfied)

^3^0–3 Likert scales (0 = not at all, 1 = several days, 2 = more than half of the days, 3 = nearly every day)

Among the 21 participants, 20 were regular users of the Care Block. Of those, 85.7% attended educational offerings, 33.3% attended physical activity classes or psychosocial offerings, 14.3% visited spaces for socializing with fellow caregivers, and 9.5% utilized care services for their dependents. Regarding regarding mobility options to access the Care Block, 42.9% walked, 28.6% used public transport, and 9.5% used informal transport (Table 1).

### Subjective Well-being

Caregivers’ average life satisfaction score was 7.0 (SD = 2.2) (Table 1). In addition, subjective well-being perception emerged as a relevant theme describing caregivers’ experience of the Care Block. Participants reported experiencing a “new sense of purpose” by engaging in the educational offering and a feeling of enjoyment in recreational activities. Moreover, by attending psychosocial support services, they reported an appreciation of their resilience (Table 2). One caregiver mentioned:

“The services offered help you a lot with personal growth, I took the office automation course, even though I don’t have a computer. We have no way to practice, but at least we learned something, we realized that we are still good at something. We are people who have had a hard time because of our age, because we come from families that didn’t have opportunities, we didn’t study or had worked since we were very young... we as housewives had very few opportunities” (Engage step participant).

**Table 2:** Examples of stakeholders’ testimonies in each Our Voice Step regarding benefits of the Care Block and the research process and barriers

Our Voice step	Quote
*Engage*	
Adequate articulation of public services and facilities	“The facilities have been very useful; the citizen services office is close enough for me to pay my bills, ask questions, and now study elementary school… I didn’t think I would study, and here I have elementary school and high school classes, systems courses, and psychosocial services” (Engage step participant)
Subjective well-being perception	“The services offered help you a lot with personal growth, I took the office automation course, even though I don’t have a computer. We have no way to practice, but at least we learned something, we realized that we are still good at something. We are people who have had a hard time because of our age, because we come from families that didn’t have opportunities, we didn’t study or had worked since we were very young… we as housewives had very few opportunities” (Engage step participant)
Mental health benefits	“I have a lot of problems, and (assisting the program) seriously one feels relief. They first help you to discharge pain, anguish, and desperation. Then they give you orientation. Besides that, they give you light” (Engage step participant)
Barriers of access	“Sometimes husbands are very jealous, and often they don’t understand you very well. They don’t like it when you go out to study and progress with something different… It is challenging because the first thing they imagine is that you are getting a lover. They don’t imagine that you are going to pick up a notebook and a pencil to learn and to be able to teach your child with a disability” (Engage step participant)
*Activate*	
Virtual Reality Experience (VRE)	“With virtual reality and the audio, I was able to observe what was actually happening… it allows you to go to the place and live it.” (Policymaker from the District Secretariat of Planning)
Overall perception of the *Activate* step	“Many times, we don’t know which doors to knock on or how to express all our needs, and many times we try to ask for help, but we don’t know how to do it. Thanks to the VRE and the project, at this moment we are communicating with the right people… we are being able to express ourselves… and now we feel heard… now we know about things that we did not know that they are moving forward (from the Government)” (Citizen scientist)
*Change*	
Strengthening of social bonds	“This process allowed us to communicate among us and with policymakers and other stakeholders. Before the process and even during it I did not fully comprehend how it helped us to get connected with other people and form social bonds among us, it was until after it finished that I realized it.” (Citizen scientist)
Value of the research process for addressing improvements of public policy	“The information community produces through this research strengthens the technical concepts we have formulated in policy. Your results overlap with our data but having them allows us to have arguments to improve some critical points in the city that hinder accessibility for its inhabitants” (Policymaker from the District Secretariat for Women)

### Mental Health Symptoms

The percentage of caregivers reporting depression and generalized anxiety symptoms were 19.0% and 23.8% respectively. In addition, mental health benefits derived from psychosocial support services emerged as a relevant theme. Participants mentioned that before attending the services, they experienced anhedonia due to caregiving responsibilities, separation from their partners, or the effects of COVID-19. The psychosocial support services allowed them to increase their cognition and emotional awareness (Table 2).

### Observed Physical Activity on the Care Block’s Terrace Area

**Fig. 3:**
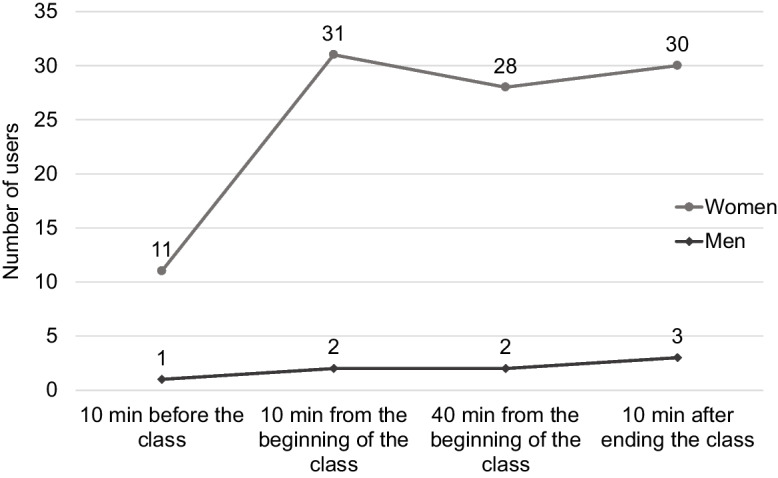
Occupation of the Care Block’s terrace by sex when physical activity classes were in place. This figure depicts the occupation of the Care Block’s terrace when physical activity classes were in place. The graph shows the number of users at four specific moments of observation. Note that at all times, female occupancy was higher than male occupancy

Compared to men, women were more likely to occupy the terrace while in physical activity classes (women: 100/108 [92.6%] versus men: 8/108 [7.4%]; *p* < 0.001). Most of the observed women (*n* = 88/101; 87.1%) engaged in moderate to vigorous physical activity classes, with aerobics being the main activity (Fig. 3). The Care Block’s terrace was characterized by a high-quality score (10 points), with most areas accessible (100%), usable (100%), supervised (93.8%), and containing organized physical activity classes (50%).

### Engage: Perceptions about the Care Block

In the *Engage* focus group session, 22 caregivers and three policy implementers from the District Secretariat for Women participated. Among the main themes that emerged, easy access to multiple services and facilities in the Care Block was frequently mentioned. Services include the citizen services office, supermarkets, care services for their dependents, and classes for caregivers. Another relevant theme concerned barriers of attendance to the program. Participants mentioned the limited support from partners, care-related overburden, income generation needs, and lack of motivation as key factors limiting their engagement in the program (Table 2).

### Discover: Discovery Tool-Identified Features Influencing Access to the Care Block

Twenty-one citizen scientists went on 42 community walks (21 from their house to the Care Block and 21 back). During their walk audits, they captured 257 photos and audio recordings. Caregivers identified facilitators (*n* = 79) and barriers (*n* = 178) to accessing the Care Block, mainly at the policy level (35.9% of comments), followed by the built environment (32.1%), social (22.6%), individual (5.7%), and interpersonal levels (3.8%) (Table 3).

**Table 3:** Facilitators and barriers of access to the Care Block ranked by themes and subthemes

**Themes** Subthemes	% (*N* = 257)	Socio-ecological level	Exemplary quote
**Care Block services and infrastructure**	**16.73% (*N* = 43)**		“What is interesting about Care Blocks is everything I have learned, much of which I had no idea about. I also had the opportunity to meet new people, some of the same age as I have and others who are older, which has allowed me to learn from their experiences. That for me has been fantastic” (Citizen scientist)
*Facilitators*	*13.62% (N = 35)*		
Education and training classes (high school, tertiary education, and skill-specific)		Policy	
Physical activity classes and infrastructure (dance, bicycle)		Policy	
Care services for recipients		Policy	
Citizen services building—SuperCade (public services payment and general citizen information)		Built environment	
Program implementers		Policy	
Sense of well-being		Individual	
*Barriers*	*3.11% (N* = *8)*		
Limited services diversification		Policy	
Care services for people with disabilities		Policy	
Low temperatures in the facilities		Built environment	
**Motorized transport**	**10.89% (*N*** = 28**)**		“‘The TransMiCable is an accessible transport, both for caregivers and care-receivers’, but if the caregiver doesn’t have money for the transport, then they cannot come (To the Care Block) because sometimes people don’t have money to pay” (Citizen scientist)
*Facilitators*	*5.44% (N* = *14)*		
Public transport		Policy	
Informal transport		Community	
Cable car (TransMiCable)		Built environment	
*Barriers*	*5.45% (N* = *14)*		
Lack of transport alternatives		Policy	
Transport occupation		Policy	
Transport price		Policy	
Travel time		Built environment	
**Enjoyment of the walk**	**3.11% (*N* = 8)**		“The good thing about this walk is that when I exercise, I have to walk a good incline. I like that every time I come here to the Care Block” (Citizen scientist)
*Facilitators*	*3.11% (N* = *8)*		
Physical activity		Individual	
Landscape		Individual	
Park availability		Built environment	
*Barriers*	*0.00%*		
**Coexistence**	**5.84% (*N* = 15)**		“She is my emotional support. I am her visual support because she is blind. We help each other to assist to the Care Block and fulfill our dream of finishing High School” (Citizen scientist)
*Facilitators*	*2.33% (N* = *6)*		
Relationships among peers		Interpersonal	
Neighbors’ solidarity		Interpersonal	
*Barriers*	*3.50% (N* = *9)*		
Limited civic culture		Community	
**Streets and sidewalks**	**29.18% (*N* = 75)**		“The person with a disability that lives on the mountain suffers a lot. To get him out of there, our caregivers experience hardship because we (two or three persons) need to carry him or her. When it’s only one of us, we need to ask the neighbors. If they are willing to help it’s good, but when they aren’t…” (Citizen scientist)
*Facilitators*	*4.28% (N* = *11)*		
Streets/sidewalks in good condition		Built environment/Policy	
*Barriers*	*24.90% (N* = *64)*		
Lack of sidewalks for care recipients		Built environment/Policy	
Mobility difficulties due to rain		Built environment	
Lack of adequate streets/sidewalks		Built environment/Policy	
Lack of paving		Built environment/Policy	
Lack of maintenance		Built environment/Policy	
Streets/sidewalks inclination		Built environment/Policy	
Sidewalk’s invasion		Community	
**Personal safety**	**15.95% (*N* = 41)**		“This is the shortcut we take, although it is a little bit dangerous on some parts because there are some drug sales and, well, the guys are more latent in some hours than others” (Citizen scientist)
*Facilitators*	0.00%		
*Barriers*	15.95% *(N* = *41)*		
Theft		Community	
Consumption/sale of psychoactive substances		Community	
Limited police presence		Policy	
Sexual violence		Community	
Poor street lighting		Built environment	
Personal safety while mobilizing on motorized transport		Community	
Personal safety while walking		Community	
**Road safety**	**7.39% (*N* = 19)**		“In this place, there is a curve where the cars go up and down, and also the cars whip around the corner. It is dangerous because no one knows whether to pass or not. When you pass, sometimes they turn very fast coming or going down” (Citizen scientist)
*Facilitators*	*0.78% (N* = *2)*		
Road safety		Policy	
*Barriers*	*6.61% (N* = *17)*		
Risks of road collisions		Built environment	
**Pollution**	**6.23% (*N* = 16)**		“This is one of the blocks where it remains dirty. Most people take out their waste ahead of time and leave it on the lookout for the streets. They leave the garbage outside so that people, or rodents, or dogs stay in the area” (Citizen scientist)
*Facilitators*	*0.00%*		
*Barriers*	*6.23% (N* = *16)*		
Animal waste		Community	
Rodents		Community	
Pollution		Community	
Garbage in the streets		Community	
**Equipment**	**4.67% (*N* = 12)**		“This space is not adequately used; it has never been functioning. I would like to know what the city is planning to do with the space. They could give someone a job for them to work here, but they have not given it” (Citizen scientist)
*Facilitators*	*1.17% (N* = *3)*		
Urban transformations		Built environment	
*Barriers*	*3.50% (N* = *9)*		
Wasted public space		Built environment	
Limited equipment		Built environment	

The most frequently mentioned facilitator was the services and facilities in the Care Block (*N* = 35/79). Citizen scientists highlighted the adequate built environment for physical activity (terrace), its class offerings (education, dance, bike lessons, and psychosocial support), and the social bonds with peers and program staff. The second most mentioned facilitator was transport (*N* = 14/79), which included formal (aerial cable cars and buses) and informal (ride-sharing taxis) options. Participants highlighted adequate quality of sidewalks and roads surrounding the Care Block as the third most mentioned facilitator (*N* = 11/79). Caregivers of the elderly enjoyed physical activity opportunities and nature while walking, as well as solidarity among their peers and neighbors to cope with their dependents’ commutes.

The most frequently mentioned barrier was the poor quality and lack of sidewalks and roads (*N* = 64/178), including the lack of road paving, absence of ramps for strollers and wheelchairs, narrow width, steepness, and impediments to sidewalk usage (garbage mismanagement and street vendors). The second most mentioned barrier was the limited perception of personal safety because of robberies, sale and consumption of psychoactive substances, and sexual violence (*N* = 41/178). Participants also identified a high risk of pedestrian injury due to the lack of sidewalks, heavy traffic, and vehicle speed as the third most mentioned barrier (*N* = 17/178). Caregivers of children identified drivers’ recklessness as a common barrier and caregivers of the elderly and people with disabilities highlighted the costs and lack of transport options.

### Visualize: Virtual Reality Experiences Accessing the Care Block

VRE allowed stakeholders to immerse themselves in simulations of real-world facilitators and barriers experienced by the caregivers. The facilitators included in the VRE were adequate sidewalks and roads, the cable car, and the Care Block services and facilities. The barriers included impediments to sidewalk usage, limited transport options, pedestrian personal safety, lack of sidewalks and roads, and high risk of pedestrian injury. A video demonstration of the VRE is available at https://youtu.be/RitnayL8hbY?si=IsrC23K-1gZeYtZg

### Activate: Actions to Collectively Improve Access to the Care Block

The stakeholder community meeting was attended by eight policymakers and implementers from Bogotá’s District Secretariat for Women (*n* = 2), Habitat (*n* = 2), Mobility (*n* = 1), and Planning (*n* = 3), along with 16 caregivers and five researchers from public health, transport, and urban planning fields. Overall, the VRE helped to improve the *Our Voice* method by allowing citizen scientists to communicate their findings and enable stakeholders to better understand caregivers’ real-world barriers (Table 2).

Citizen scientists suggested urgent infrastructure improvements, fines for garbage mismanagement, and speed reducers. The representative from the District Secretariat of Habitat mentioned their organization is developing strategies to support better access to the Care Blocks and is implementing tactical urbanism interventions to enhance the public space. To address personal safety concerns, participants proposed police cameras, 24/7 attention commands, and peer groups for walking.

Discussions with stakeholders also underscored the limited access to the Care Block for people with disabilities. Participants suggested transport subsidies and a free bus program to enhance accessibility for people with disabilities. A representative from the District Secretariat for Women mentioned ongoing efforts to explore strategies that increase the involvement of people with disabilities in the program. The discussion evoked additional concerns for people with disabilities like limited access to public services and subsidies in general. A representative from the District Secretariat of Planning recommended consulting with local disability councils and generating petitions as forms of citizen participation to demand specific rights.

The experience served as a call for co-responsibility among all stakeholders to become involved in specific actions, including (i) focusing on the urgent needs in the built environment, along with continued participation in dialogues between diverse stakeholders; (ii) recognizing the rights of community members to be involved in citizen participation; and (iii) involving researchers in the further development of guidelines that provide feedback on public policy interventions (Table 2).

The survey results from the community meeting attendees indicated that they considered the VRE to be easy or moderately easy to use (67%) and enhanced the *Our Voice* method by allowing participants to communicate the barriers accessing the Care Block more clearly (76%). Stakeholders and caregivers expressed that VRE could help create innovative solutions (81%) and facilitate the dialogue between them (71%).

### Change: Ongoing Dialogues

We documented short-term improvements in local capacity building and strengthening of social bonds after the stakeholder meeting. A citizen scientist reported that participating in the research encouraged a support network between some participants. She further expressed interest in leveraging her leadership to work toward the well-being of caregivers in her community (Table 2).

Moreover, the research team developed a services web page guide and shared it with the 16 citizen scientists who attended the *Activate community meeting*. Participants found the stakeholder meeting and the subsequent guide beneficial for understanding city-level programs. Some even reported attending those services (e.g., services offered by the District’s Superior Education, Science, and Technology Agency).

The District Secretariat of Planning policymakers (*n* = 6) (Table 2) highlighted both the *Our Voice* method and VRE as powerful tools to provide the local community a leading role in the research-to-action process. District Secretariat of Habitat (*n* = 5) and Women (*n* = 2), and Ciudad Bolivar Local Mayors’ Office (*n* = 1) policymakers agreed that this community-based research allowed them to better understand how to enhance the Care Block program from a bottom-up approach (Table 2). In sharing the study results at the Smart Cities Expo Bogotá 2023, we found that attendees perceived VRE as a way to tour Ciudad Bolívar, understand the access barriers experienced in caregiver's everyday lives, and observe how the Care Block can be beneficial for them.

## Discussion

In this study, citizen scientists indicated that the Care Block program is improving their well-being through service provision. Participants highlighted the facilities and services provided (citizen services office, supermarkets, services for care recipients, and classes) as facilitators promoting their engagement in the program. Notably, participation in physical activity classes was higher among women. However, the poor quality and lack of sidewalks and roads were persistent barriers hindering the program’s accessibility, especially for people with disabilities. The VRE promoted engaging dialogue between stakeholders and participants, supporting community advocacy aimed at improving built environment features and community-led strategies. Overall, the results reinforce residents’ support of the Care Block as an innovative, integral urban care program.

The global agenda’s proposed actions to recognize, reduce, and redistribute unpaid caregiving require services targeting the dyad caregiver-recipient [25]. However, the literature introducing the potential relationship of policy-level care programs on women caregivers’ well-being and mental health symptoms remains scarce [26]. The results of our study are in part consistent with a study conducted in the UK that shows a negative association between unpaid caregiving and subjective well-being that widens over time [27]. Similarly, a study conducted among unpaid caregivers in Bogotá reported that participants associated physical activity classes in the Care Blocks with increased self-care activities, improved corporal mobility, and reduced fatigue, stress, and isolation [8]. Our study adds to the current evidence highlighting that educational and psychosocial services could foster caregivers’ sense of purpose, enjoyment, resilience, and cognitive and emotional awareness. This is particularly crucial for a population with low levels of well-being. When comparing the subjective well-being survey results from our study with national estimates, we found participants reported on average, life satisfaction levels 1.2 points lower than the average for Colombian women [28]. The low levels of well-being could be related to the fact that 75% of our participants spend more than 9 h per day on unpaid care work. Higher time spent on unpaid caregiving could be related to time poverty for other activities such as income generation, studying, and leisure activities. Furthermore, the results regarding depression and generalized anxiety symptoms are similar to the overall population of Bogotá (19.95% and 27.03% of Bogotá’s women report depression and generalized anxiety symptoms respectively) [29].

Our study also revealed that Care Block participants face access challenges, which is in line with feminist urbanism [30] and mobility of care literature [31]. Because of unpaid care work, women travel more across the city, use the bus more often, and walk more than men [31]. Therefore, transport interventions are key for reducing the time women spend on unpaid care work. Citizen scientists indicated that transport programs (e.g., free program buses, subsidies) are urgent to enhance access to the Care Block. Likewise, they underscored the importance of community-based strategies to address access barriers (e.g., peer groups for walking), which is consistent with previous *Our Voice* studies exemplifying that community action in tandem with policy interventions can lead to positive changes [11].

Additionally, this study adds to the *Our Voice* literature by integrating VRE into the citizen science-based method. The VRE simulation allowed for a deeper understanding of the transformative effect that the urban environment might have by facilitating empathy towards unpaid caregivers’ experiences and leveraging advocacy processes. Previous studies have shown that virtual reality enhances the communicative process within decision-making related to security perception [32], street walkability [33], and the adequation of public spaces [34]. While VRE has been mentioned as a potential next step in *Our Voice* projects [35], our study is the first of its kind to integrate it into the method.

This study has some methodological limitations that should be considered. First, there was a limited sample size for *Our Voice* given that participants were mainly users of the first Care Block in Bogotá. However, prior *Our Voice* studies have shown that a sample as small as eight participants can activate local changes beneficial to the larger community [10]. With that said, future studies should consider including participants who cannot access the program to get a more comprehensive perspective. Second, this study implemented a short-term follow-up due to time constraints but may have missed longer-term “ripple effects” [11]. We are aware that subjective well-being outcomes should be tracked over a longer period than our study allowed. Future studies should consider this.

In conclusion, understanding from a socio-ecological approach that the social and physical environment can influence potential subjective well-being benefits derived from the Care Block provides valuable insights into urban care recommendations. First, intersectoral public policy articulation is key to synergistically deliver services for caregivers. Second, Care Blocks have the potential to be high-impact policy interventions with the power to reshape an urban network that enables care work recognition. Third, implementing an urban care system that connects unpaid caregivers with opportunities for personal growth can bring positive social outcomes, such as the emergence of leadership and networks of support among program users. Fourth, adopting urban care as a comprehensive policy requires leveraging empathy and understanding local needs through community-led advocacy processes using innovative strategies that bring together stakeholders.

## Supplementary Information

Below is the link to the electronic supplementary material.

Supplementary file1 (PDF 4691 KB)

## Data Availability

The consent form signed by participants stated that data can only be shared among researchers. For future studies we will ask participants if we can share data with the journals.
