# Supplementary material for: Urban Care for Unpaid Caregivers: Community Voices in the Care Block Program, in Bogotá, Colombia
Source: J Urban Health. 2024 Sep 24;101(6):1113–27. doi: 10.1007/s11524-024-00899-z (PMC11652459; doi:10.1007/s11524-024-00899-z)
Supplement: Supplementary file 1 — Supplementary file1 (PDF 4691 KB) [file 11524_2024_899_MOESM1_ESM.pdf]

Supplementary material 1: Methodology to develop The Virtual Reality Experience

The Virtual Reality experience (VRE) was developed through a 3-step process:

1. 360-degree images and ambient sound: Following the Our Voice data analysis and first community meeting, we prioritized the caregiver's most frequently visited sites. Then, we pinpointed the three most mentioned themes (consisting of both facilitators and barriers), which were documented during the *Discover* and *Discuss* steps. To decide on the hotspot locations, we used the Discovery Tool portal to reference the geotagged photos that citizen scientists captured. During a site visit in October 2023, we took pictures of the selected themes using a 360-degree GoPro camera and recorded ambient sounds using an iPhone 10. We included 20 locations, captured 76 photos, and recorded corresponding ambient sounds from each respective location. After reviewing the photos, we filtered them down to 15 locations and grouped 3 photos (with different themes; Table 1) each into five different VRE "paths."

Table 1. 360 photos included in the Virtual Reality Experience (VRE) and their corresponding theme.

Path	Photo	Theme(s)
1	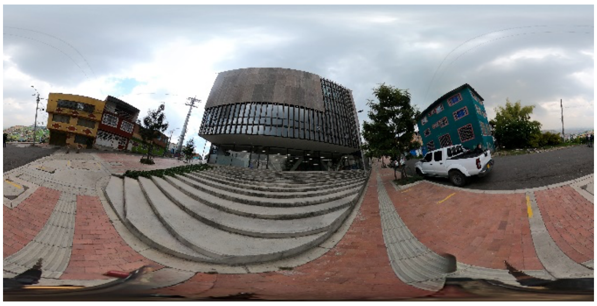	Care Block's services and infrastructure
	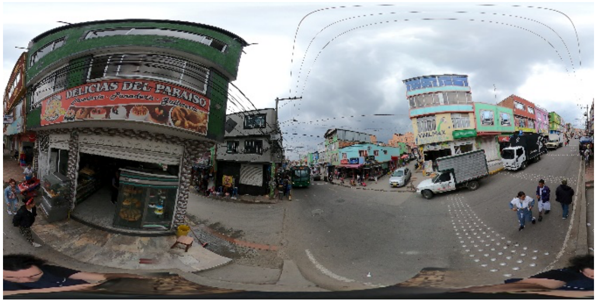	Transport Occupation

	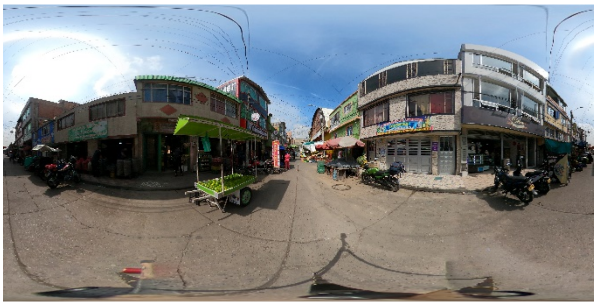	Sidewalks invasion
2	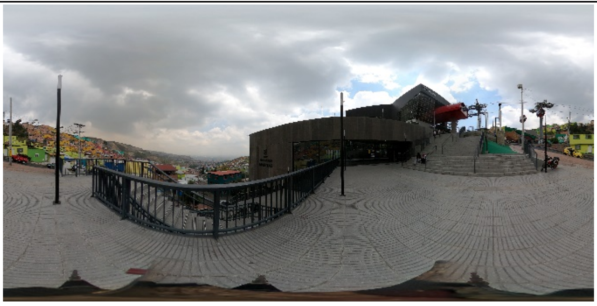	Citizen services building - SuperCade
	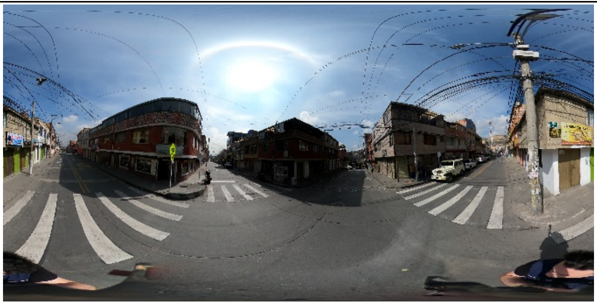	Informal transport
	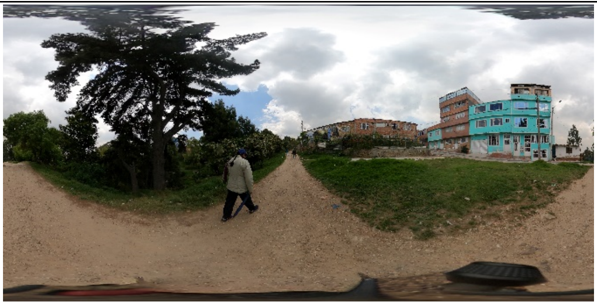	Lack of paving<br>insecurity
3	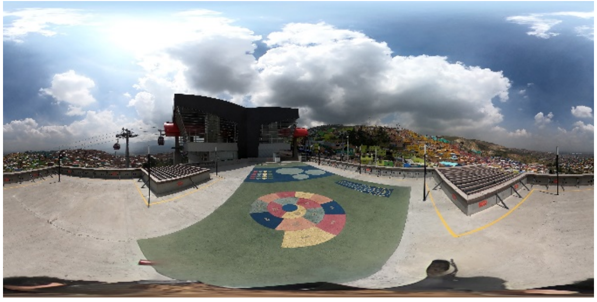	Physical activity infrastructure

	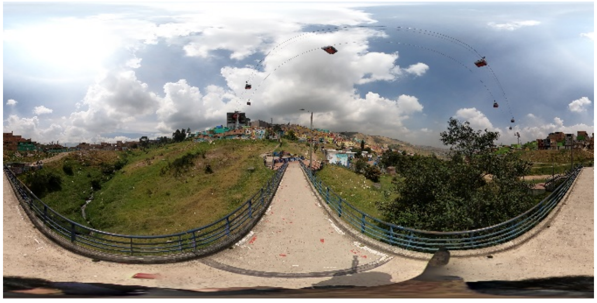	Insecurity
	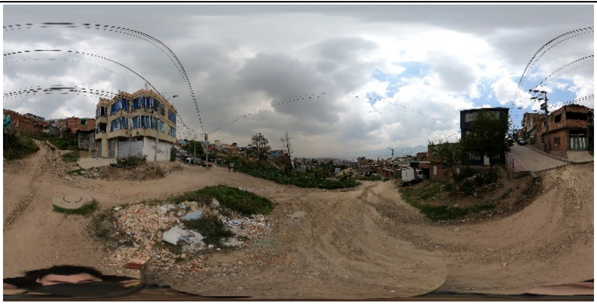	Lack of paving<br>Lack of sidewalks for dependents
4	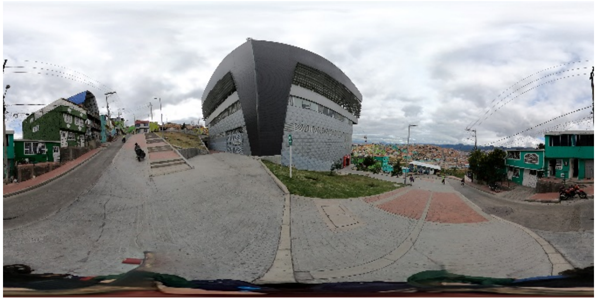	Streets/sidewalks in good condition
	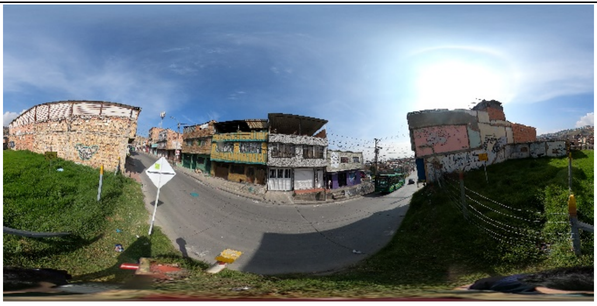	Risks of road collisions<br>Lack of adequate streets/sidewalks
	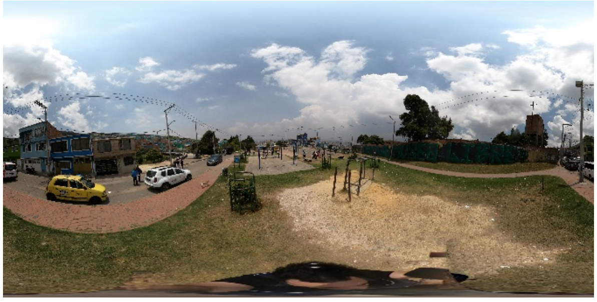	Consumption/sale of psychoactive substances

5	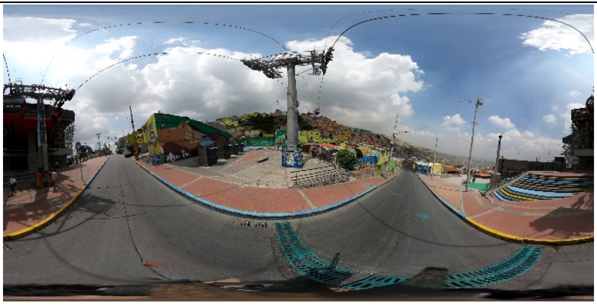	Risks of road collisions<br><br>Lack of sidewalks for dependents
	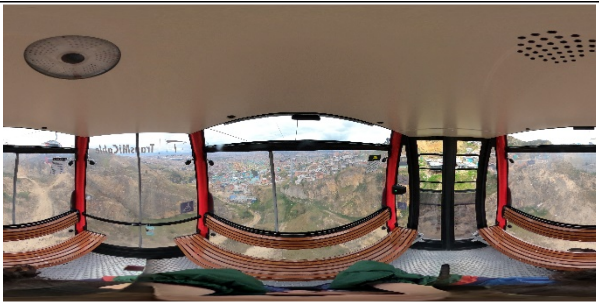	Cable car service (TransMiCable)
	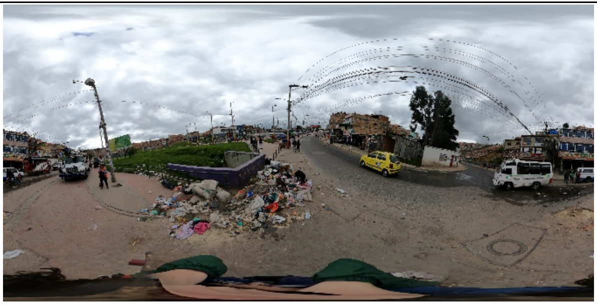	Garbage in the streets

2. Experience design: The VRE was developed using the Unity game engine (2021.3.11f1 version) and simulated using the Oculus Quest 2 Virtual Reality headset. Unity's platform allowed for the integration of 360-degree visualization, as well as game interactivity, which allowed users the ability to change between while experiencing the simulation. To accomplish this immersive experience, each image was projected on the internal surface of a sphere (Figure 1). The user point-of-view was set to the center of this sphere, allowing them to visualize the 360-degree image by pivoting around their standing position while wearing the Oculus headset. 3D virtual structures resembling buttons (Figure 2) were concurrently presented to the user. These virtual buttons worked as interactable objects whose function was to change the 360 images projected inside the sphere. The user was able to navigate each button using the Oculus controllers. 3D representations of each Oculus controller (left and right) were included in the VRE (Figure 2) to familiarize community members with the virtual scenario. In addition to button interactivity, corresponding ambient sounds were automatically projected to users via built-in headphones in the Oculus headset.

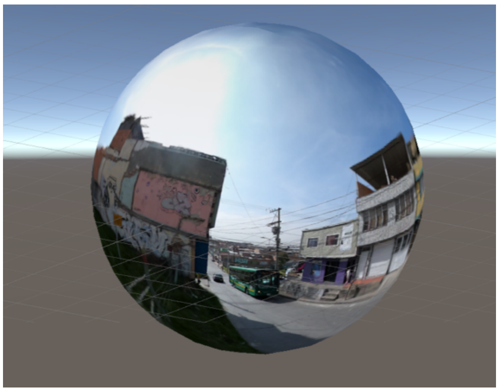

Figure 1. Projection of a 360-degree image of a location inside a sphere for the creation of the immersive experience in Unity.

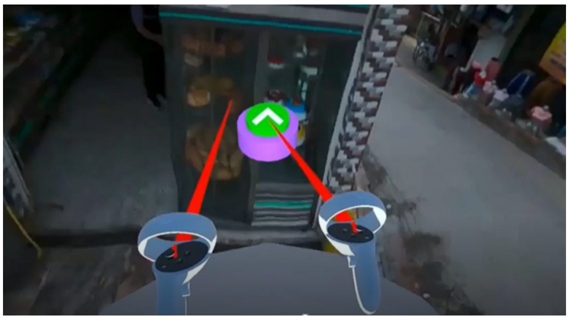

Figure 2. Virtual button and an example of the button interaction using 3D representations of Oculus controllers in VRE.

Community members´ voice recordings were included in the VRE using happy and sad characters as interactable objects (Figure 3). Using the characters promoted an effortless method to associate each commentary with a facilitator (happy face) or a barrier (sad face). The user was able to hear the recorded comment by interacting with the button using Oculus controllers. Additionally, each character was strategically placed near the object of interest (e.g., a street, a sidewalk, or a bus from public transport), reflecting the theme documented by the citizen scientist.

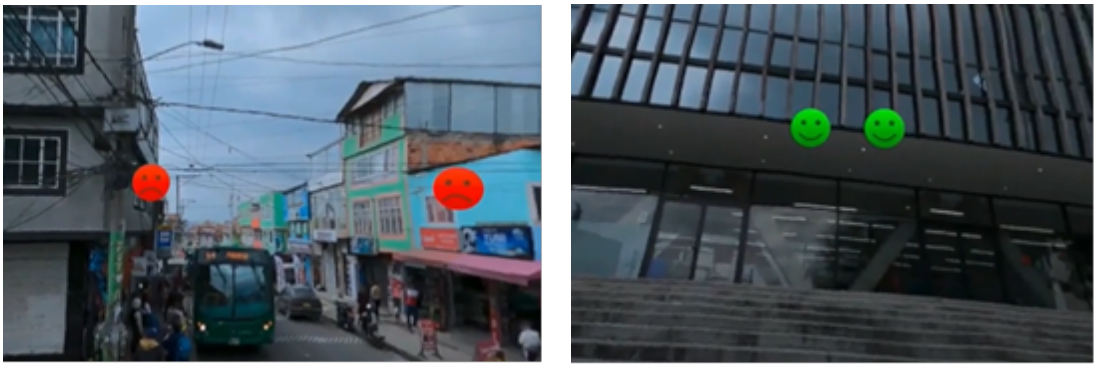

Figure 3. Interactable emojis (sad and happy faces) are used to reproduce commentary audios of the community members.

Ultimately, we designed an immersive experience that begins with five buttons marked for the user to select the desired path (Figure 4). Each pathway corresponds to three images that users could change using the interactive button.

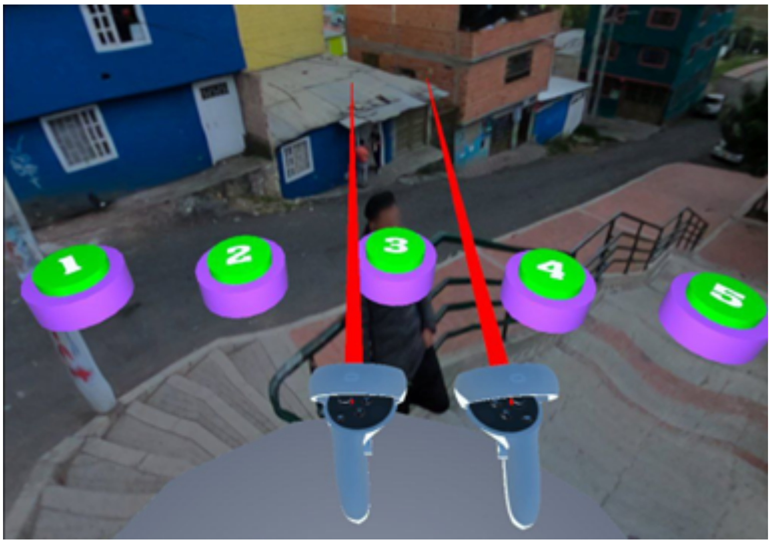

Figure 4. Example of the interactive selection of the five pathways in the VR experience.

3. Citizen scientists training: After designing the experience, in February 2023, we trained five citizen scientists who participated in the second community meeting on how to use the VRE. This process consisted of familiarization with the Oculus headset and controller functionality. Each pathway was randomly assigned to each participant so they could explore the locations and hear the comments on the themes. Ultimately the training allowed the citizen scientists to explain and guide the policymakers through the entire VRE during the following step of the OV process.

Supplementary material 2: Results facilitators and barriers of access to the Care Block with pictures.

Table 2. Facilitators and barriers of access to the Care Block ranked by themes and subthemes, Our Voice for Caregivers in the Care Block.

Themes<br>**Subthemes**	**%**<br>**(N = 257)**	**Socio-ecological level**	**Exemplary quote**	**Photo**
Care Block services and infrastructure<br>*Facilitators*	**16.73%**<br>**(N=43)**<br>*13.62%*<br>*(N=35)*		"*What is interesting about MDC is everything I have learned, much of*	

Education and training classes (high school, tertiary education, and skill-specific)		Policy	*which I had no idea about. I also had the opportunity to meet new people, some of the same age as I have and others who are older, which has allowed me to learn from their experiences. That for me has been fantastic*” (Citizen scientist).	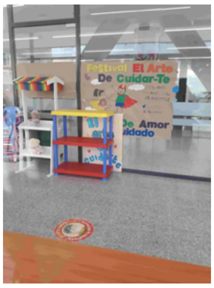
Physical activity classes and infrastructure (dance, bicycle)		Policy		
Care services for recipients		Policy		
Citizen services building - SuperCade (public services payment and general citizen information)		Built environment		
Program implementers		Policy		
Sense of well-being		Individual		
Barriers	*3.11% (N=8)*			
Limited services diversification		Policy		
Care services for people with disabilities		Policy		
Low temperatures in the facilities		Built environment		
Motorized transport	**10.89% (N=28)**		“*'The TransMiCable is an accessible transport, both for caregivers and care-receivers', but if the caregiver doesn't have money for the transport, then they cannot come (To the Care Block) because sometimes people don't have money to pay*” (Citizen scientist).	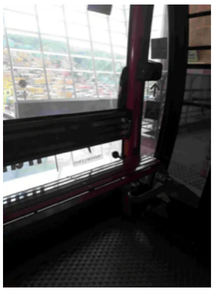
Facilitators	*5.44% (N=14)*			
Public transport		Policy		
Informal transport		Community		
Cable car service (TransMiCable)		Built environment		
Barriers	*5.45% (N=14)*			
Lack of transport alternatives		Policy		
Transport occupation		Policy		
Transport price		Policy		
Travel time		Built environment		
Enjoyment of the walk	**3.11% (N=8)**		"*The good thing about this walk is that when I exercise, I have to walk a good incline. I like that every time I come here to the Care Block*" (Citizen scientist).	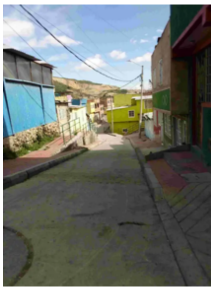
Facilitators	*3.11% (N=8)*			
Physical activity		Individual		
Landscape		Individual		
Park availability		Built environment		
Barriers	*0.00%*			
Coexistence	**5.84% (N=15)**		“*She is my emotional support. I am her visual support because she is blind. We help each other to assist to the Care Block and*	
Facilitators	*2.33% (N=6)*			
Relationships among peers		Interpersonal		
Neighbors’ solidarity		Interpersonal		

Barriers	*3.50% (N=9)*		*fulfill our dream of finishing High School*" (Citizen scientist).	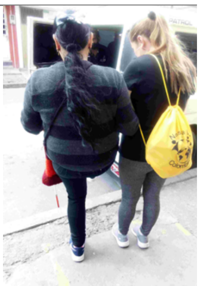
Limited civic culture		Community		
Streets and sidewalks	**29.18% (N=75)**		"*The person with a disability that lives on the mountain suffers a lot. To get him out of there, our caregivers experience hardship because we (two or three persons) need to carry him or her. When it's only one of us, we need to ask the neighbors. If they are willing to help it's good, but when they aren't…"* (Citizen scientist).	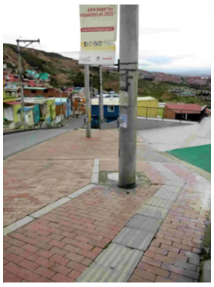
Facilitators	*4.28% (N=11)*			
Streets/sidewalks in good condition		Built environment/Policy		
Barriers	*24.90% (N=64)*			
Lack of sidewalks for care recipients		Built environment/Policy		
Mobility difficulties due to rain		Built environment		
Lack of adequate streets/sidewalks		Built environment/Policy		
Lack of paving		Built environment/Policy		
Lack of maintenance		Built environment/Policy		
Streets/sidewalks inclination		Built environment/Policy		
Sidewalk's invasion		Community		
Security	**15.95% (N=41)**		"*This is the shortcut we take, although it is a little bit dangerous on some parts because there are some drug sales and, well, the guys are more latent in some hours than others*" (Citizen scientist).	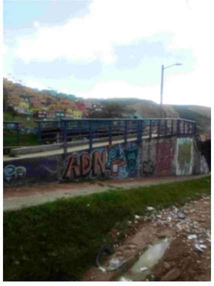
Facilitators	0.00%			
Barriers	15.95% *(N=41)*			
Theft		Community		
Consumption/sale of psychoactive substances		Community		
Limited police presence		Policy		
Sexual violence		Community		
Poor street lighting		Built environment		
Personal safety while mobilizing on motorized transport		Community		

Personal safety while walking		Community		
Road Safety	**7.39% (N=19)**		"*In this place, there is a curve where the cars go up and down, and also the cars whip around the corner. It is dangerous because no one knows whether to pass or not. When you pass, sometimes they turn very fast coming or going down*" (Citizen scientist).	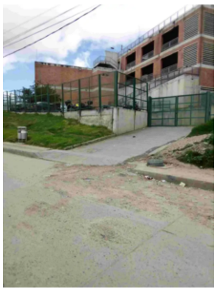
Facilitators	*0.78% (N=2)*			
Road safety		Policy		
Barriers	*6.61% (N=17)*			
Risks of road collisions		Built environment		
Pollution	**6.23% (N=16)**		"*This is one of the blocks where it remains dirty. Most people take out their waste ahead of time and leave it on the lookout for the streets. They leave the garbage outside so that people, or rodents, or dogs stay in the area*" (Citizen scientist).	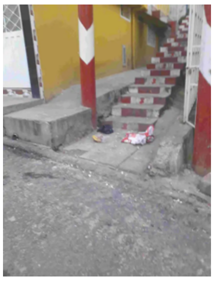
Facilitators	*0.00%*			
Barriers	*6.23% (N=16)*			
Animal waste		Community		
Rodents		Community		
Pollution		Community		
Garbage in the streets		Community		
Equipments	**4.67% (N=12)**		"*This space is not adequately used; it has never been functioning. I would like to know what the city is planning to do with the space. They could give someone a job for them to work here, but they have not given it*" (Citizen scientist).	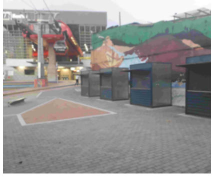
Facilitators	*1.17% (N=3)*			
Urban transformations		Built environment		
Barriers	*3.50% (N=9)*			
Wasted public space		Built environment		
Limited equipment		Built environment		
